# The Association between Late Third-Trimester Oxytocin Level and Early-Onset Postpartum Depression Symptoms among Jordanian Mothers: A Cross-sectional Study

**DOI:** 10.1155/2022/7474121

**Published:** 2022-02-14

**Authors:** Hasan Rawashdeh, Zahra Alalwani, Amer Sindiani, Rana Alodetalah, Mohammad Alqudah

**Affiliations:** ^1^Department of Obstetrics and Gynaecology, Jordan University of Science and Technology (JUST), Post Office Box 3030, Irbid 22110, Jordan; ^2^King Abdulla University Hospital (KAUH), Ar-Ramtha, Jordan, Post Office Box: 630001, Irbid, 22110, Jordan; ^3^Department of Obstetrics and Gynecology at King Abdulla University Hospital (KAUH), Ar-Ramtha, Post Office Box: 630001, Irbid, 22110, Jordan; ^4^Department of Pathology and Microbiology, Jordan University of Science and Technology (JUST), Post Office Box 3030, Irbid 22110, Jordan

## Abstract

**Purpose:**

Oxytocin has been suggested to play a vital role in modulating maternal behavior and stress-related disorders. However, the relationship between antenatal oxytocin and postpartum depression is not well established. We aim to investigate the association between serum oxytocin level in the late third-trimester and early-onset postpartum depression symptoms.

**Materials and Methods:**

A total of 172 healthy pregnant women participated in this cross-sectional descriptive study. The serum oxytocin level was measured between 34 and 37 weeks. A validated Edinburgh Postnatal Depression Scale (EPDS) was used to assess symptoms of depression four to six weeks postpartum. Participants who scored more than 12 on the EPDS were considered having depressive symptoms. Independent sample *t*-test and Pearson *r* were used to examine differences in depression scores. The level of significance was set at *α* = 0.05.

**Results:**

30.8% of the participants experienced depressive symptoms. There was no association between EPDS scores and oxytocin level *r*(170) = 0.10, *p* = 0.23. The association also did not exist even among women with a lifetime history of depression *r*(43) = −0.13, *p* = 0.37. Participants with low education, low income, previous history of depression, positive family history of depression, positive family issues, and absent emotional family support have scored significantly higher on EPDS scores than their counterparts. The strongest association was with previous lifetime history of depression *t*(170) = −4.40, *p* < 0.001.

**Conclusions:**

Postpartum depression is a major public health problem in Jordan. Late trimester serum oxytocin level has no association with early-onset postpartum depression.

## 1. Introduction

Pregnancy is a major life event that is inevitably accompanied by social, psychological, and hormonal changes. In fact, from a psychological point of view, it is considered the most complex event in human experience. Pregnancy can trigger a wide range of emotions from transient mood liability to severe depressive episodes [1].

Traditionally, postpartum psychiatric disorders (PPPD) have been classified into postpartum blues, postpartum psychosis, and postpartum depression. Postpartum depression (PPD) is the most common form and is defined as a depressive episode, starting within 6 months after childbirth, that meets the DSM-IV criteria for a major depressive episode, without psychotic features [2].

In Jordan, the incidence of PPD is about 22%, which is comparable to the nearby countries [3–5]. It places PPD as one of the most common and most serious public health problems since it is a major contributor to the 8% of maternal deaths related to mental health problems [6]. Moreover, infants of PPD mothers are at significant risk of physical, emotional, cognitive, and interpersonal problems in their later lives [1].

PPD is often underdiagnosed and untreated for many reasons related to maternal underreporting of symptoms, poor awareness of the medical care providers, and limited sensitivity of the available screening tools [7]. On the other hand, there is good evidence showing that PPD can be prevented if prophylactic antidepressants were commenced immediately after delivery for the high-risk population [8]. This has urged researchers to explore possible associations between PPD and specific antenatal biomarkers that have the potential to predict and identify PPD objectively before symptoms develop.

Many biomarkers were studied like oxytocin level, leptin level, brain-derived neurotrophic factor (BDNF), and luteinizing hormone : follicular stimulating hormone ratio, but the oxytocin level is the best-studied brain system in humans with vast growing evidence showing its effect on social perception, behavior, and social memory, the amygdala, and stress-related disorders [9–14].

Oxytocin is a peptide hormone synthesized in the supraoptic and paraventricular nuclei of the hypothalamus and released into the bloodstream via the posterior pituitary gland. Due to the prosocial effects of oxytocin along with its antidepressant and anxiolytic properties, we decided to test its association with PPD. In this study, we aim to investigate the association between serum oxytocin concentration in the late third-trimester and postpartum depression in the early postnatal period among Jordanian mothers.

## 2. Materials and Methods

In this cross-sectional descriptive study, the participants were sampled from the antenatal clinics at King Abdulla University Hospital (KAUH) which is the largest tertiary hospital in the north of Jordan. During the regular late antenatal visits, every pregnant woman attending the clinics was interviewed by a designated interviewer where a full description of the study design was delivered. The interviewer was a medical practitioner who collected a brief socioeconomic history and provided instant answers for the candidates' inquiries before completing the written informed consent of participation if they have fulfilled the inclusion criteria and agreed to participate. All candidates were allowed to withdraw from the study at any time.

The sample size was computed utilizing the G∗ Power software [15]. A power level of 0.80, an alpha level of 0.05, and an effect size of 0.5 for the independent *t*-test were used to calculate the sample size. The estimated sample size was 128 based on the previous parameters. The study's final sample size was 172 participants.

### 2.1. Subjects

The participants were mothers having a singleton pregnancy, between 34 and 37 weeks of gestation, not known to have chronic medical illnesses nor current mental illnesses, not known to have fetal abnormalities whether growth, structural, or chromosomal, and no history of neonatal deaths for the current pregnancy. Both primgravid and multigravida women were included. Gestational age was confirmed by reviewing the first-trimester scan report between 11 and 14 weeks using the crown rump length (CRL) measurement.

**Figure 1:**
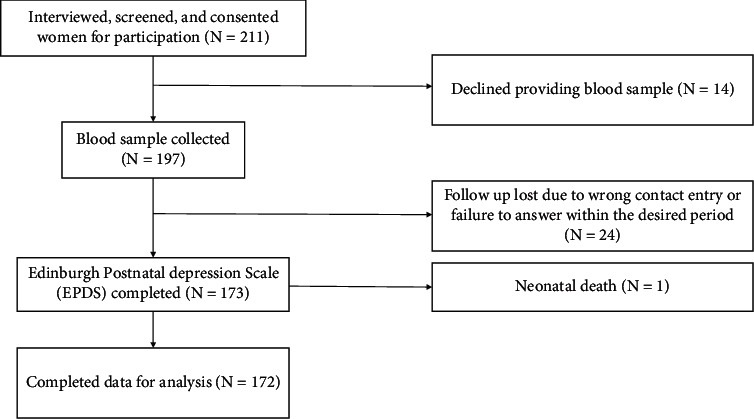
Flowchart of study participants.

Over 16 months' duration, starting in September 2018 and ending by December 2019, 211 participants were admitted to the study. 14 of them declined to provide a blood sample and asked to be withdrawn. 197 blood samples were collected. Follow-up was lost with 24 participants because they provided the wrong contact details or they failed to answer within the specific period. There was one case of early neonatal death after one week of delivery where the entry was removed. The final complete sample was consisted of 172 participants where they completed the consent of participation, provided blood sample for serum oxytocin concentration, and answered our phone call after 4 to 6 weeks of their delivery of a live singleton newborn and completed the Edinburgh Postnatal Depression Scale (EPDS) by the same interviewer. A flowchart of participants is displayed in Figure 1.

EPDS is the most commonly used validated screening tool for postpartum depression [16, 17]. A score of 13 and above is the most widely used score to define probable depression [18]. A validated translation of EPDS into the Arabic language was used to assess postpartum depression symptoms [17]. A total of 10 items, dealing with typical PPD symptoms, were answered on a 4-point scale. Women who scored 13 and above were considered positive for depression.

Ethical approval was gained by the IRB committee (Reference number: 48/116/2016 on 31/05/2018). Demographic and clinical data were collected from the medical records, while socioeconomic data were completed during signing the consent. All participants had completed and signed the informed consent for participation.

### 2.2. Sample Collection and Storage

All experimental work took place at the research laboratory located at the same hospital. All safety protocols were applied to all procedures. Biohazardous materials were disposed of according to the biosafety guideline regulations.

Samples were collected by the Vacutainer technique into one plain tube used to separate serum for enzyme-linked immunosorbent assay (ELISA) for oxytocin. All samples were collected between 11 : 00 and 15 : 00. Samples were centrifuged at 4000 rpm for 7-10 minutes. Serum from plain tubes was transferred into 2 clearly labeled Eppendorf tubes. All aliquots were stored in a deep freezer at -80°C.

### 2.3. Enzyme-Linked Immunosorbent Assay (ELISA)

Preparation of reagents, standards, diluents, and buffers took place before starting the ELISA procedure exactly as mentioned in the kits' manual. The competitive ELISA technique was conducted using kits from Ray Biotech company (Georgia, USA). Series of seven consecutive steps were done using the fully automated ELISA device “Elisys Uno” by HUMAN company (Wiesbaden, Germany).

First, microwell strips were prepared at room temperature. Second, assay diluent B, antioxytocin antibody, standards, and samples were prepared. After that, the antioxytocin antibody was added to all wells and incubated for 1.5 hours at room temperature. Next, the wells were washed with washing buffer four times to remove excess amount of antibodies followed by adding standards and samples to their assigned positions and incubated for 2.5 hours at room temperature. The fifth step included washing the wells again with washing buffer four times to remove excess amount of standards and samples; then, Streptavidin-HRP was added and incubated for 45 minutes at room temperature. Then, all wells were washed with washing buffer four times to remove excess amount of Streptavidin-HRP, and TMB substrate solution was added and incubated for 30 minutes at room temperature avoiding exposure to direct light. Finally, a stop solution was added to all wells and read immediately on a microwell ELISA reader spectrophotometer using 450 nm as the primary wavelength, before absorbance readings underwent a series of mathematical equations to generate a standard curve and calculate sample results.

### 2.4. Statistical Analysis

Data were analyzed using the Statistical Package for Social Science (SPSS) version 23 (SPSS, Inc, Chicago, IL). Descriptive statistics including mean (*M*), standard deviation (SD), and frequency (%) were utilized to describe the sample. The independent sample *t*-test was used to examine differences in depression scores based on selected demographic, psychosocial, and clinical variables. Pearson product-moment correlation was used to assess the relationship between EPDS and age, maternal age, and oxytocin serum concentration. The level of significance for all analyses was set at *α* = 0.05.

## 3. Results

### 3.1. Sample Characteristics

The mean age for the participants was 31 (SD = 4.96) ranging from 18 to 41 years old. The majority of participants were well educated as more than three-quarters of them had a bachelor's degree or above (*n* = 134). Although half of the participants were employed (*n* = 86), most of them had a total family monthly income of less than 1000 Jordanian dinars (≈1400 US dollars) (*n* = 128). More than half of the participants gave birth to a newborn of the female gender (*n* = 91).

The EPDS was normally distributed where the skewness level was 0.1. Fifty-three participants have scored 13 or above at the EPDS revealing a prevalence of PPD among our cohort to be 30.8%. The mean score of participants at EPDS was 9.98 (SD = 5.13), ranging from 0 to 24.

About a quarter (26%, *n* = 45) had a previous history of depression, while only 4% had a family history of depression (*n* = 7). The majority of participants (90%, *n* = 155) felt that they had adequate family support, and another 90% were satisfied by the health care services received during their stay in the hospital. Only 3.5% of the sample had family issues (*n* = 6).

Seventy-six percent of the pregnancies were planned (*n* = 131). Almost one-fifth of the sample were primgravid (*n* = 34). The mean gestational age of delivery was 37.8 (SD: 1.31) ranging from 35 to 41 weeks. About half of the deliveries were elective caesarean section (*n* = 85), 12.7% were emergency caesarean section (*n* = 22), and 37% were vaginal deliveries (*n* = 64). Postpartum haemorrhage complicated six deliveries.

Among the 64 participants who delivered vaginally, 79% did not receive any medication for pain management. Episiotomy and repair of perineal tears were performed for 15% (*n* = 26) and 26% (*n* = 44) of the sample, respectively.

The mean serum oxytocin concentration was 195.10 ng/ml (SD = 123.62) ranging from 10.38 to 498.78. The mean concentration for participants who scored less than 13 was 184.16, while it was 219 for participants who scored 13 or more. The difference between the means of oxytocin concentration for both groups was not significant, as the *t*-test illustrated.

Results of the sample characteristics are presented in Table 1.

No missing or outlier values were detected in the analyzed data.

### 3.2. EPDS Scores and Serum Oxytocin Concentration

Pearson *r* indicated that there was a nonsignificant direct correlation between EPDS scores and serum oxytocin concentration. On the other hand, there was a nonsignificant inverse correlation *r*(43) = −0.13, *p* = 0.37 between the EPDS scores and the serum oxytocin concentration among women with a lifetime history of depression, as shown in Table 2.

### 3.3. EPDS Scores and Selected Psychosocial and Clinical Variables

As illustrated in Table 3, the independent *t*-test has shown that there was a statistically significant difference *t*(170) = −4.40, *p* < 0.001, between the mean EPDS score of participants with a previous lifetime history of depression (*M* = 12.73, SD = 4.76) and EPDS score of participants without previous history of depression (*M* = 9.01, SD = 4.91). Also, there was a statistically significant association between the mean EPDS score and the level of education, level of income, family history of depression, family issues, and emotional family support.

Participants with low education, low income, previous history of depression, positive family history of depression, positive family issues, and absent emotional family support have scored significantly higher on EPDS score than their counterparts.

Table 2 demonstrates that there was no relationship between EPDS scores with the gestational age of delivery nor maternal age using Pearson *r* test.

## 4. Discussion

Postpartum depression (PPD) continues to represent a major public health problem. Many studies were carried out to define the possible etiologic factors behind it. Some have suggested psychosocial factors like poverty, low social support, pain, stress, and anxiety to play a major role [19], while other studies have focused more on the biochemical background suggesting neuroendocrinological factors like Estradiol and oxytocin to be causative factors [12, 20]. The rest of the studies have highlighted the role of the genetic predisposition and the family history of depression [21].

Due to the available evidence showing the link between oxytocin with maternal behavior, mother-infant bonding, and stress-related disorders along with its antidepressant and anxiolytic properties, we have established this study primarily to test the association between oxytocin and PPD [14, 22]. At the same time, we have studied the associations between PPD and other psychosocial and clinical variables as potential etiologic factors suggested in the literature [19].

Unlike our proposed assumption, we found no significant association between antenatal serum oxytocin concentration in the late third-trimester (34 to 36 weeks) and early postpartum depression symptoms (4 to 6 weeks). Hence, isolated late trimester oxytocin concentration is not a useful marker for early-onset PPD. On the other hand, there was a statistically significant association between PPD and below bachelor's degree level of education, below 1000 Jordanian dinars (≈1400 US dollars) family monthly income, positive family history of depression, positive family issues, previous history of depression, and absent emotional family support. Therefore, it is recommended to include these variables during screening process for PPD, with special focus on the presence of a previous history of depression.

Regarding the association with oxytocin, our results partially agreed with Massey et al. who at first found no correlation between antenatal oxytocin and PPD symptoms. But, a direct significant association appeared only among women with a lifetime history of depression [23]. In fact, when we tested oxytocin with postpartum depression among women with a lifetime history of depression, similar to [23], we found a nonsignificant inverse correlation.

In comparison with [12], our results were not similar. Their study showed a significant inverse correlation between antenatal oxytocin and PPD symptoms. They demonstrated that plasma oxytocin level significantly predicted PPD symptoms 2 weeks after delivery. Also, they found a significant correlation between oxytocin level and gestational age at delivery [12]. Although the settings of the study carried out by [12] were very similar, we did not find the inverse correlation between oxytocin and PPD symptoms, nor we found any relation between oxytocin and gestational age at delivery. This variance might be attributed to the difference in the number of participants (74 : 172), the difference in the ethnic groups of participants (Switzerland : Jordan), the difference of the cut-point levels of EPDS (10 : 13), and the difference in the time of assessment of PPD symptoms (2 weeks : 4 to 6 weeks).

In comparison with Eapen et al., they ended up finding no correlation between antenatal oxytocin and postpartum depression, similar to our results. However, a significant inverse correlation was drawn between serum oxytocin 3 months after delivery with antenatal and postnatal depression [24].

Indeed, the available results from our work and the aforementioned works have generated a more complicated picture of the association between antenatal oxytocin and PPD. This conclusion was previously suggested by two recent systematic reviews by Moura et al. and Thul et al. testing the association between antenatal and postnatal oxytocin concentration with PPD symptoms, where they have found inconclusive results [25, 26]. Therefore, we are proposing some possible available theories that might be able to explain the disintegration between peripheral oxytocin level and its effect centrally. First, Cyranowski et al. suggested that peripheral oxytocin may have a dysregulated pattern of release in depressed women making it unreliable [27]. Second, the genetic variation in the oxytocin receptor gene may play a role in modulating the response of different candidates to the same stimuli, as suggested by Chen et al. and Jonas et al. [28, 29]. Third, there may be different signals that alter the oxytocin physiological response. This theory was suggested by Stubue et al. after they found that breastfeeding had intensified the relationship between oxytocin and PPD [30].

Regarding the association of PPD with psychosocial factors, in comparison between our study and a previous study that was conducted in the north of Jordan, as well, we ended up with similar results in terms of the significant association between PPD with low income and poor family emotional support.

Our study has highlighted the alarming prevalence of PPD among Jordanian mothers sitting at 30.8%. Furthermore, 13 participants (out of 172, representing 7.5%) have admitted that they had thoughts of harming themselves at different frequencies. One of them had these thoughts quite often.

### 4.1. Limitations

Although this study is the first-of-its-kind that tested the association between PPD and antenatal serum oxytocin concentration among Middle Eastern women, and the study sample used was the biggest, there were some limitations in the study. The first limitation is that the sample was collected from a single hospital where most women descended from the same ethnic, social, and environmental background, which makes it difficult to generalize the findings on all women. The second limitation is that EPDS has limited sensitivity and specificity [31]. Future researchers are encouraged to combine oxytocin levels with other biological, psychosocial, or clinical variables before testing its association with postpartum depression. The third limitation is that we have not assessed the change in oxytocin level (postpartum level compared with antenatal level) which is more important than a single antenatal value. Future studies are recommended to assess the association between oxytocin transition and PPD.

## 5. Conclusions

About one in three women in Jordan suffers from postpartum depression. Therefore, active interventions should be implemented immediately including antenatal and postnatal screening for PPD.

There was no association between late antenatal third-trimester serum oxytocin level and early-onset postpartum depression symptoms. A significant association was found between PPD and low educational level, low income, positive family history of depression, positive family issues, previous history of depression, and absent emotional family support, making these variables essential during screening for PPD. The association between PPD and previous lifetime history of depression was noticeable.

## Figures and Tables

**Table 1:** Sample characteristics for the participants. Mean: *M*; standard deviation: SD; %: percent (*N* = 172).

Variable	*N* (%)
Demographic variables	
Age	172 (100)
Employment	
Employed	86 (50)
Housewife	86 (50)
Income	
Low	128 (74.70)
High	044 (25.60)
Gender of the baby	
Male	81 (47.10)
Female	91 (52.90)
Level of education	
School	25 (14.50)
Diploma	13 (07.60)
Bachelor	121 (70.30)
Master	11 (06.40)
Philosophy degree	2 (01.20)
Psychosocial variables	
Edinburgh Postnatal Depression Scale (EPDS)	172 (100)
EPDS score	
<13	119 (69.20)
≥13	053 (30.80)
Previous history of depression	
Yes	045 (26.20)
No	127 (73.80)
Family history of depression	
Yes	007 (04.10)
No	165 (95.90)
Family emotional support	
Yes	155 (90.10)
No	017 (09.90)
Family issues	
Yes	006 (03.50)
No	166 (96.50)
Satisfied with health care	
Yes	155 (90.10)
No	007 (09.90)
Clinical variables	
Serum oxytocin concentration (ng/ml)	172 (100)
Gestational age (weeks)	
Planned pregnancy	
Yes	131 (76.20)
No	041 (23.80)
Parity	
Prim gravid	034 (19.80)
Multigravida	138 (80.20)
Presence history of miscarriages	
Yes	047 (27.30)
No	125 (72.70)
Mode of delivery	
Caesarean section	108 (62.80)
Vaginal delivery	064 (37.20)
Analgesics use during labour	
Yes	036 (56.25)
No	028 (43.75)
Episiotomy	
Yes	026 (40.62)
No	038 (59.38)
Perineal suturing	
Yes	044 (68.75)
No	020 (31.25)
Postpartum haemorrhage	
Yes	006 (03.50)
No	166 (96.50)
Gestational diabetes	
Yes	002 (01.20)
No	170 (98.80)
Induced labour	
Yes	013 (07.60)
No	159 (92.40)

**Table 2:** Pearson product-moment correlation to assess the correlation between Edinburgh Postnatal Depression Scale (EPDS) scores with maternal age, serum oxytocin concentration, and gestational age of delivery (*N* = 172).

Variable	EPDS scores *r*	*p* value
Maternal age	0.02	0.76
Oxytocin serum concentration	0.10	0.23
Gestational age of delivery	0.05	0.54

**Table 3:** Independent *t*-test to examine the difference in Edinburgh Postnatal Depression Scale (EPDS) based on selected demographic, social and psychological, and clinical variables (*N* = 172).

Variable	*M* (SD)	*t*(170)	*p* value
Employment		1.20	0.23
Employed	09.51 (5.09)		
Nonemployed	10.45 (5.15)		
Income		2.17	0.03
Low	10.48 (4.95)		
High	08.55 (5.42)		
Gender of the newborn		1.08	0.27
Male	09.53 (4.78)		
Female	10.38 (5.41)		
Education		2.50	0.01
Below bachelor	12.32 (5.46)		
Bachelor and above	09.59 (4.98)		
Previous history of depression		-4.40	<0.001
Yes	12.73 (4.76)		
No	09.01 (4.91)		
Family history of depression		-2.45	0.01
Yes	14.57 (5.25)		
No	9.79 (5.05)		
Family emotional support			
Yes	9.71 (5.02)	2.12	0.03
No	12.47 (5.60)		
Family issues			
Yes	15.50 (5.71)	-2.73	0.007
No	9.78 (5.01)		
Satisfied with health care		-0.73	0.46
Yes	10.08 (5.12)		
No	09.12 (5.27)		
Planned pregnancy		1.49	0.13
Yes	09.66 (5.01)		
No	11.02 (5.43)		
Parity		1.81	0.07
Primgravid	08.56 (4.56)		
Multigravida	10.33 (5.22)		
Previous history of miscarriages		-0.46	0.64
Yes	10.28 (5.05)		
No	09.87 (5.17)		
Mode of delivery		-1.04	0.29
Caesarean section	10.30 (5.36)		
Vaginal delivery	9.45 (4.70)		
Postpartum haemorrhage		-0.65	0.51
Yes	11.33 (5.27)		
No	09.93 (5.13)		
Induced labour		0.99	0.31
Yes	08.62 (5.47)		
No	10.09 (5.10)		

## Data Availability

The data that support the findings of this study are available from King Abdulla University Hospital. Restrictions apply to the availability of these data, which were used under license for this study. Data are available from Hasan Rawashdeh with the permission of the IRB committee at King Abdulla University Hospital.
